# Supplementary material for: Defining Kinetic Properties of HIV-Specific CD8+ T-Cell Responses in Acute Infection
Source: Microorganisms. 2019 Mar 4;7(3):69. doi: 10.3390/microorganisms7030069 (PMC6462943; doi:10.3390/microorganisms7030069)
Supplement: Supplementary file 1 [file microorganisms-07-00069-s001.pdf]

Defining kinetic properties of HIV-specific CD8$^+$ T-cell responses in acute infection

Yiding Yang and Vitaly V. Ganusov

Supplementary Information

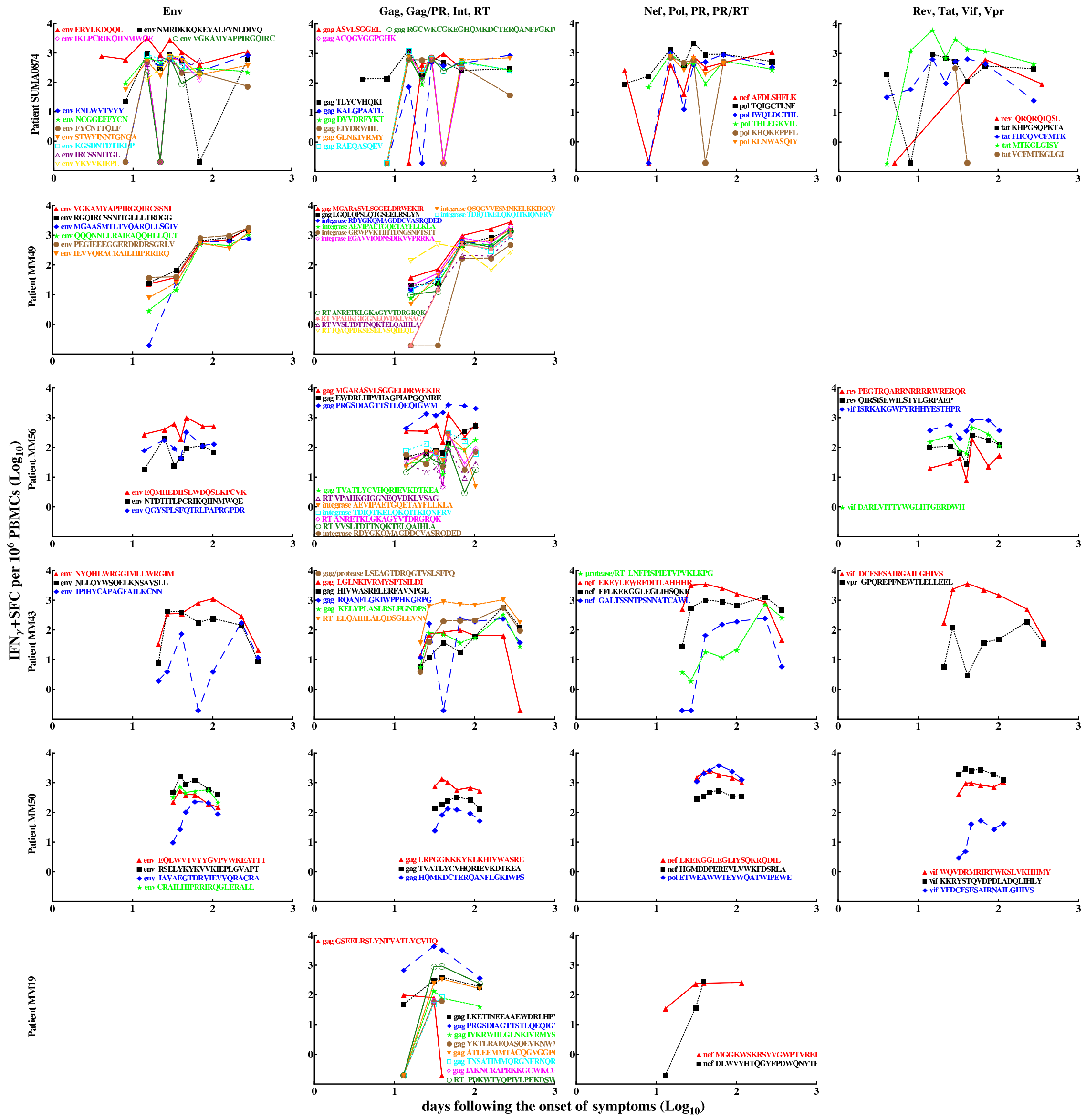

Figure S1: Kinetics of HIV-specific CD8$^+$ T-cell responses measured by IFN-γ ELISPOT assay in patients SUMA0874, MM49, MM56, MM43, MM50, and MM19. Measurements below the level of detection are plotted as having a value of 0.1. Patients are listed in descending order according to the total number of T-cell responses measured. We divided T cell responses into four groups according to their target protein (1: Env; 2: Gag, Gag/Protease, Integrase, RT; 3: Nef, Pol, Protease, Protease/Rt; 4: Rev, Tat, Vif, Vpr).

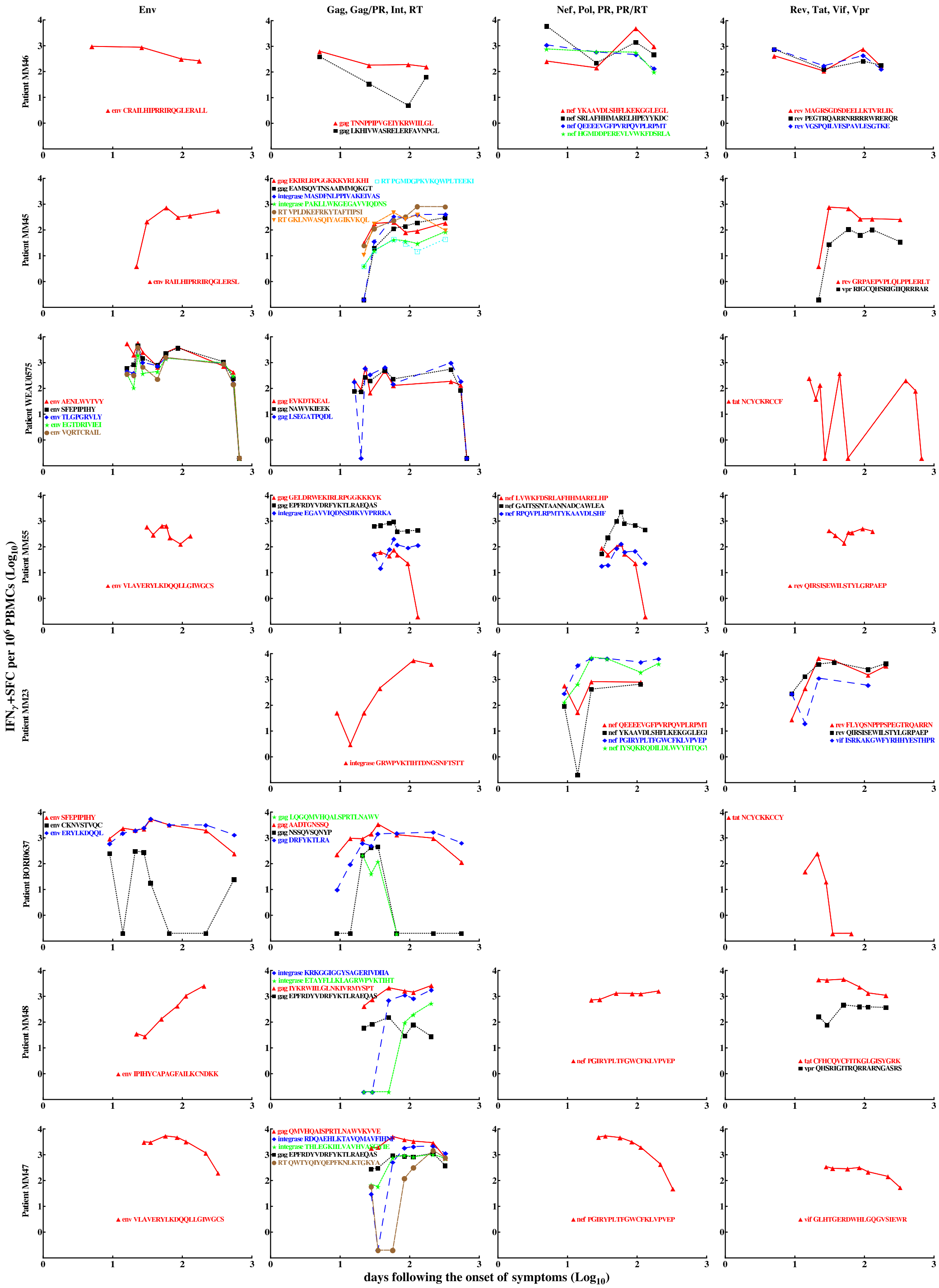

Figure S2: Kinetics of HIV-specific CD8$^+$ T-cell responses measured by IFN-γ ELISPOT assay in patients MM46, MM45, WEAU0575, MM55, MM23, BORI0637, MM48, and MM47. See Fig. S1 caption for more detail.

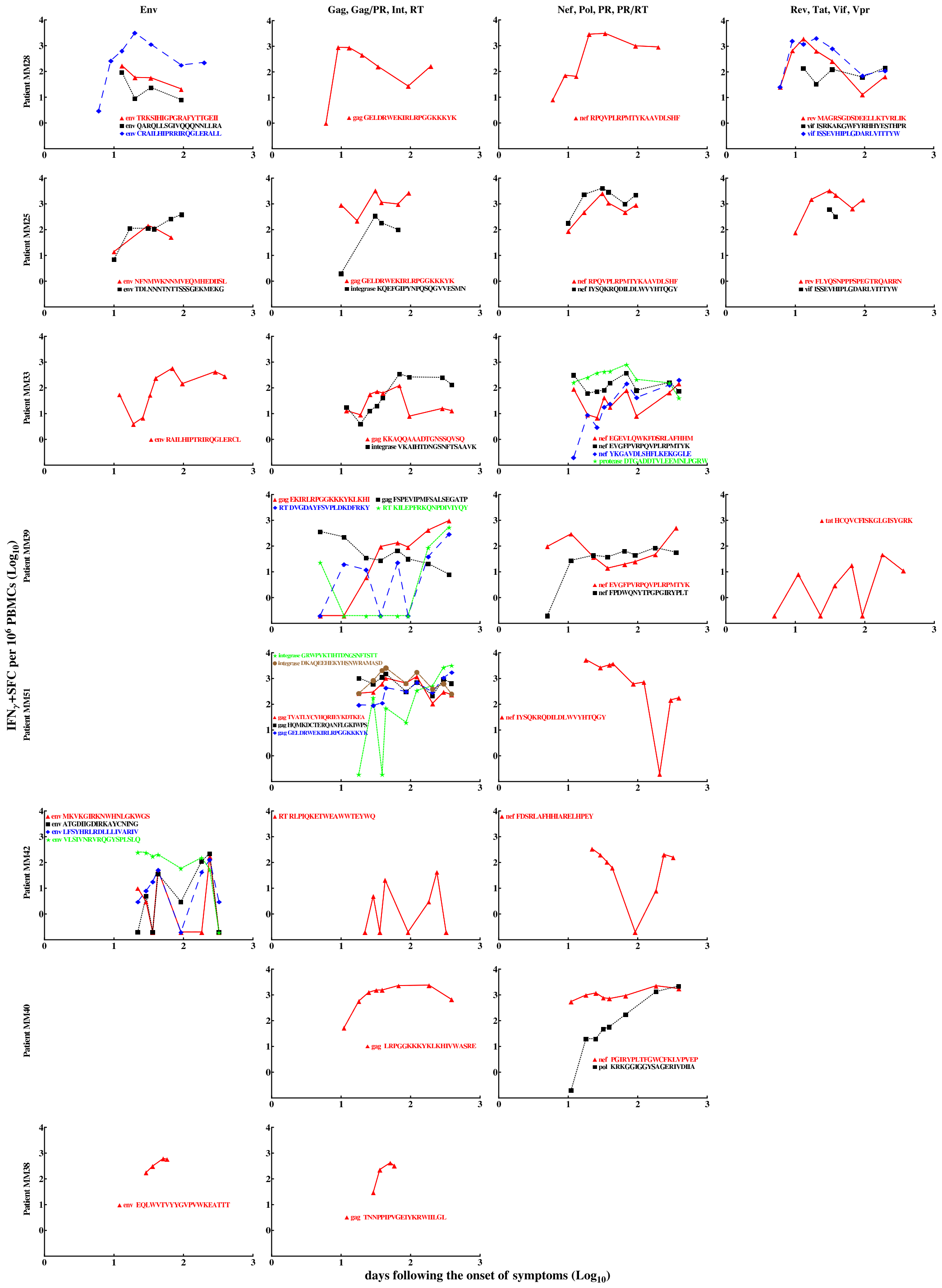

Figure S3: Kinetics of HIV-specific CD8$^+$ T-cell responses measured by IFN-γ ELISPOT assay in patients MM28, MM25, MM33, MM39, MM51, MM42, MM40, and MM38, respectively. See Fig. S1 caption for more detail.

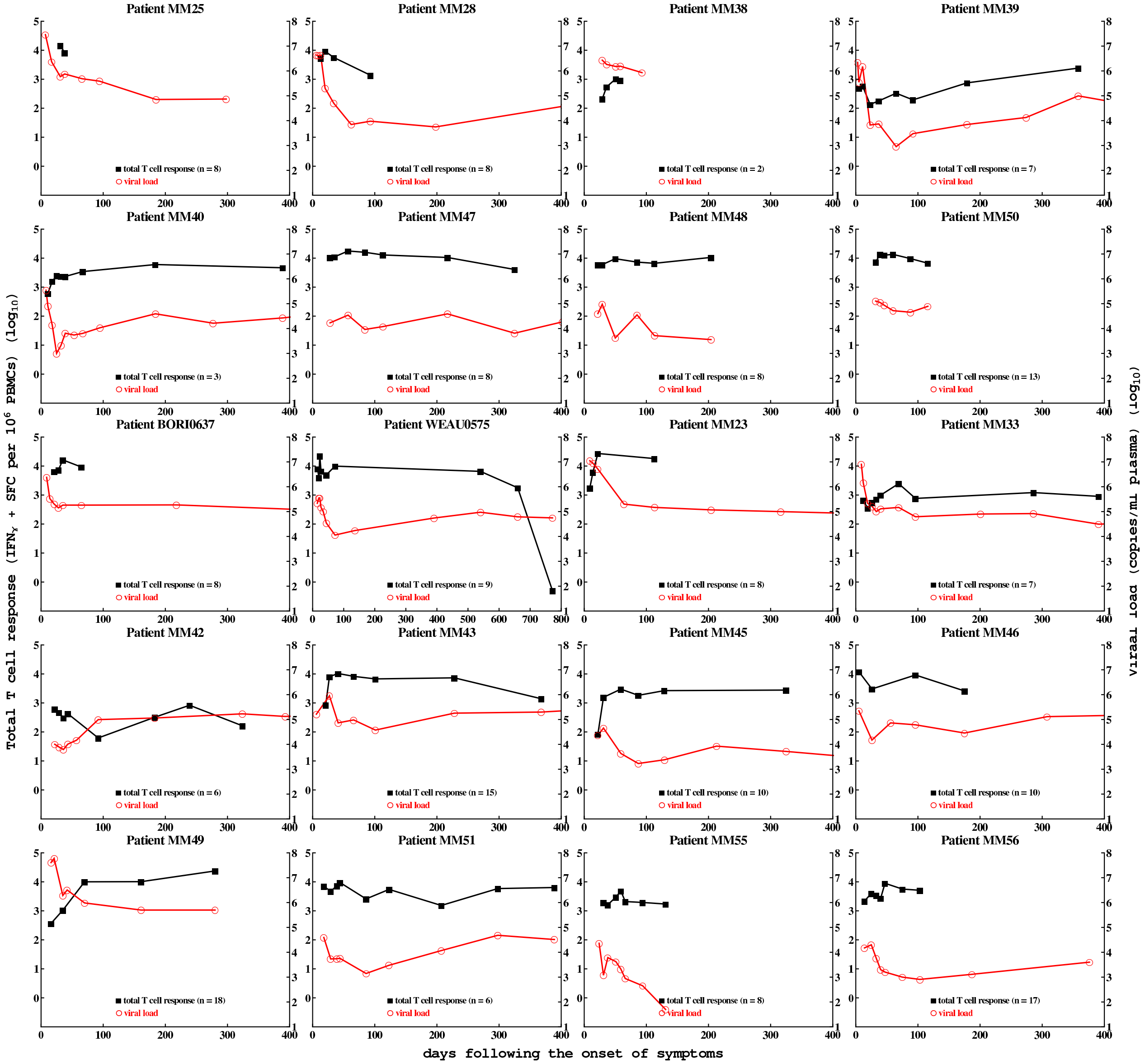

Figure S4: Kinetics of total HIV-specific CD8$^+$ T-cell response measured by IFN-γ ELISPOT assay and viral load in 20 patients in the cohort. For each patient, total CD8$^+$ T-cell response (squares) and viral load (circles) are plotted over time. Note that patient WEAU0575 was followed for longer than all other patients (772 days after symptom onset). Patients SUMA0874 and MM19 were excluded from this plot due to insufficient measurements of all T-cell responses at all time points.

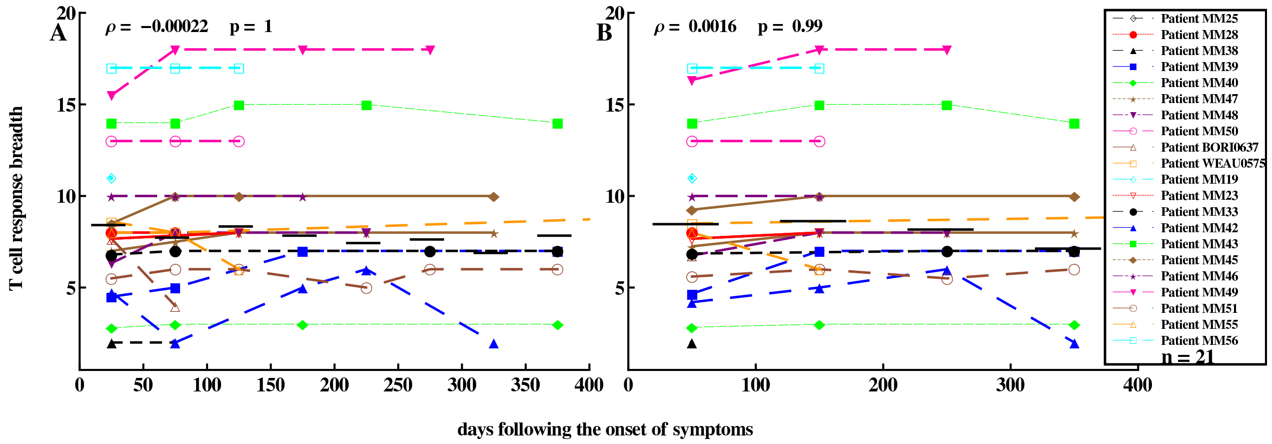

Figure S5: Nonsignificant change in the number of HIV-specific CD8$^+$ T cell responses in all patients over the course of infection. We divided the whole observation period into different time bins (50-day intervals (A) or 100-day intervals (B)) and calculated the number of T-cell responses (breadth) for the corresponding group. Small horizontal bar denotes mean breadth for that time interval. Spearman's rank coefficient was used to determine the significance of breadth change over time (correlation coefficient ρ and p values). Patient SUMA0874 was excluded from this plot due to insufficient measurements in all T-cell responses at all time points.

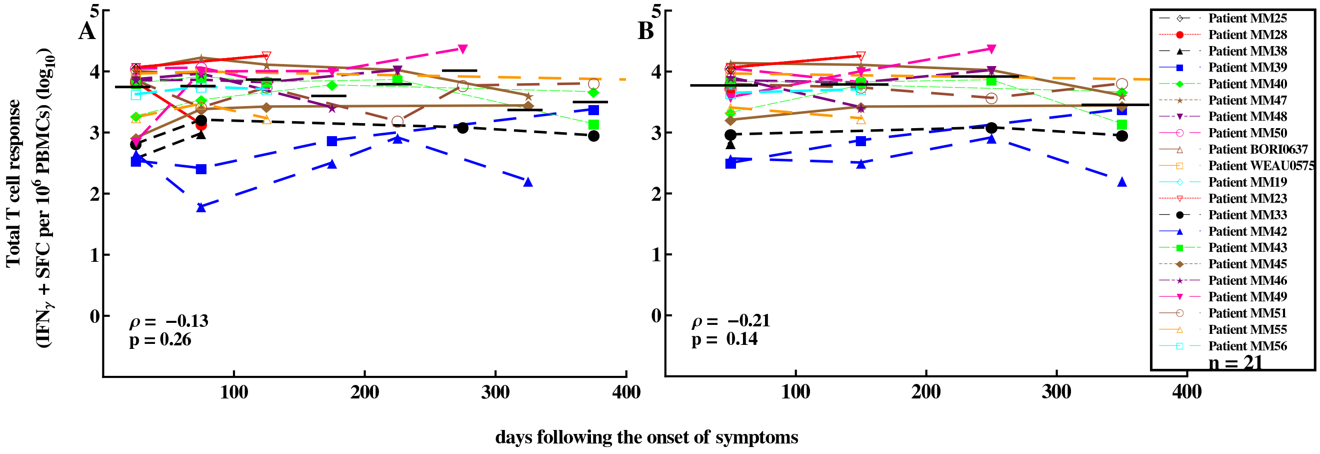

Figure S6: Nonsignificant changes of total CD8$^+$ T-cell response in all patients. We divided the whole observation period into different time bins (50-day intervals (A) or 100-day intervals (B)) and calculated the sum of all T-cell responses for a given patient. Small horizontal bar denotes average CD8$^+$ T cell response level for that time interval. For different time intervals (e.g., 15- or 30-day intervals), we found similar trends (results not shown).

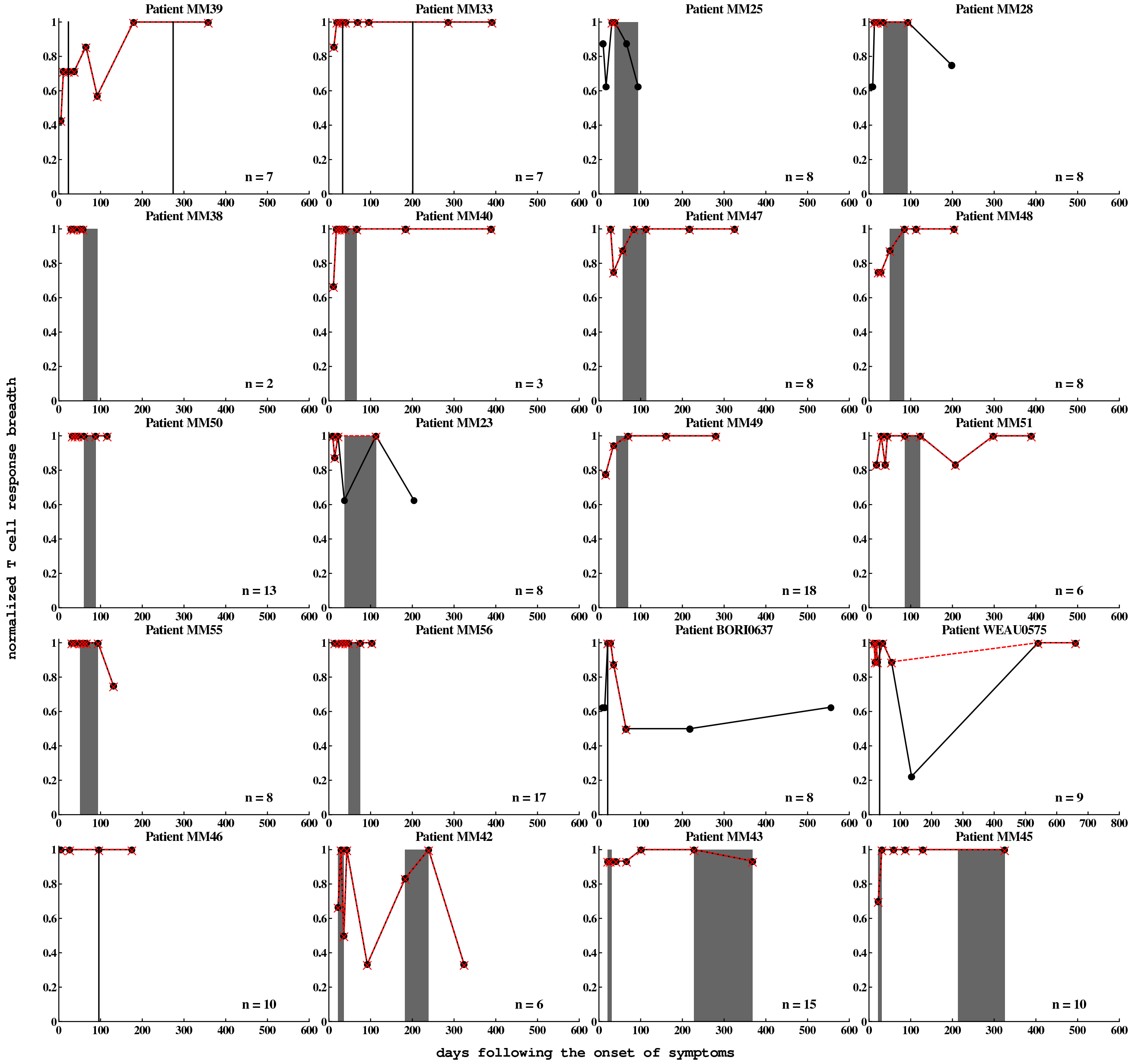

Figure S7: Variable dynamics of T-cell response breadth in individual patients. Normalized immune response breadth was defined as the number of responses at a particular time divided by the total number of responses measured in that patient. The shaded bars (or vertical lines) denote times when T-cell response mapping was performed with pooled PBMCs in each patient; in patients MM33 and MM39, mapping was performed twice. Due to missing measurements ("nd") in some epitope-specific CD8$^+$ T cell responses, we estimated the breadth at certain time points for a particular patient in two ways: 1) ignoring the time point (red crosshair ×), or 2) replacing the "nd" with 0 (black dot •) when there was at least one missing measurement at this time point. We found that in some patients (e.g., MM45, MM48, MM49) the breadth expanded slightly to saturation level, and in others, contraction phases followed the saturation (e.g., MM43, MM55). Patient WEAU0575 was followed for 772 days after symptom onset, so the x-axis for this patient is longer. Patients SUMA0874 and MM19 were excluded from this plot due to insufficient measurements of all T-cell responses at all time points.

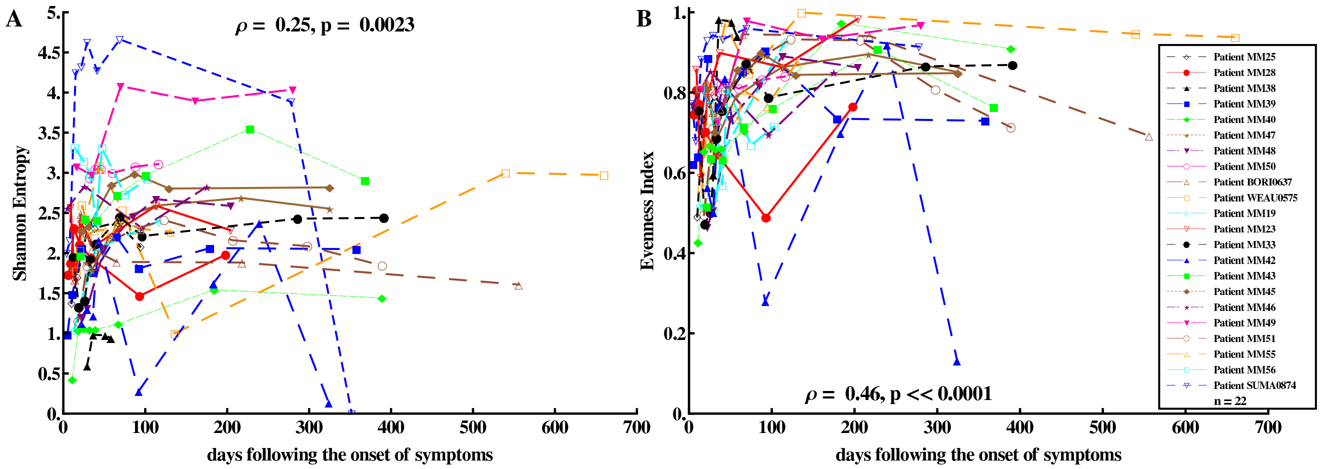

Figure S8: SE and EI of T-cell responses moderately increased over time. SE and EI were calculated at different time points for all patients (see Material and Methods for more detail); we found a moderate but statistically significant positive trend (Spearman Rank Correlation: $\rho = 0.30$ ($p = 0.00074$) and $\rho = 0.49$ ($p \ll 0.0001$)). Major significant changes in both measures of breadth occurred within the first 40 days of symptom onset. Analyses included only the time points at which all CD8$^+$ T-cell responses were measured. Detailed SE and EI kinetics in each patient are shown in Figs. S9 and S10, respectively.

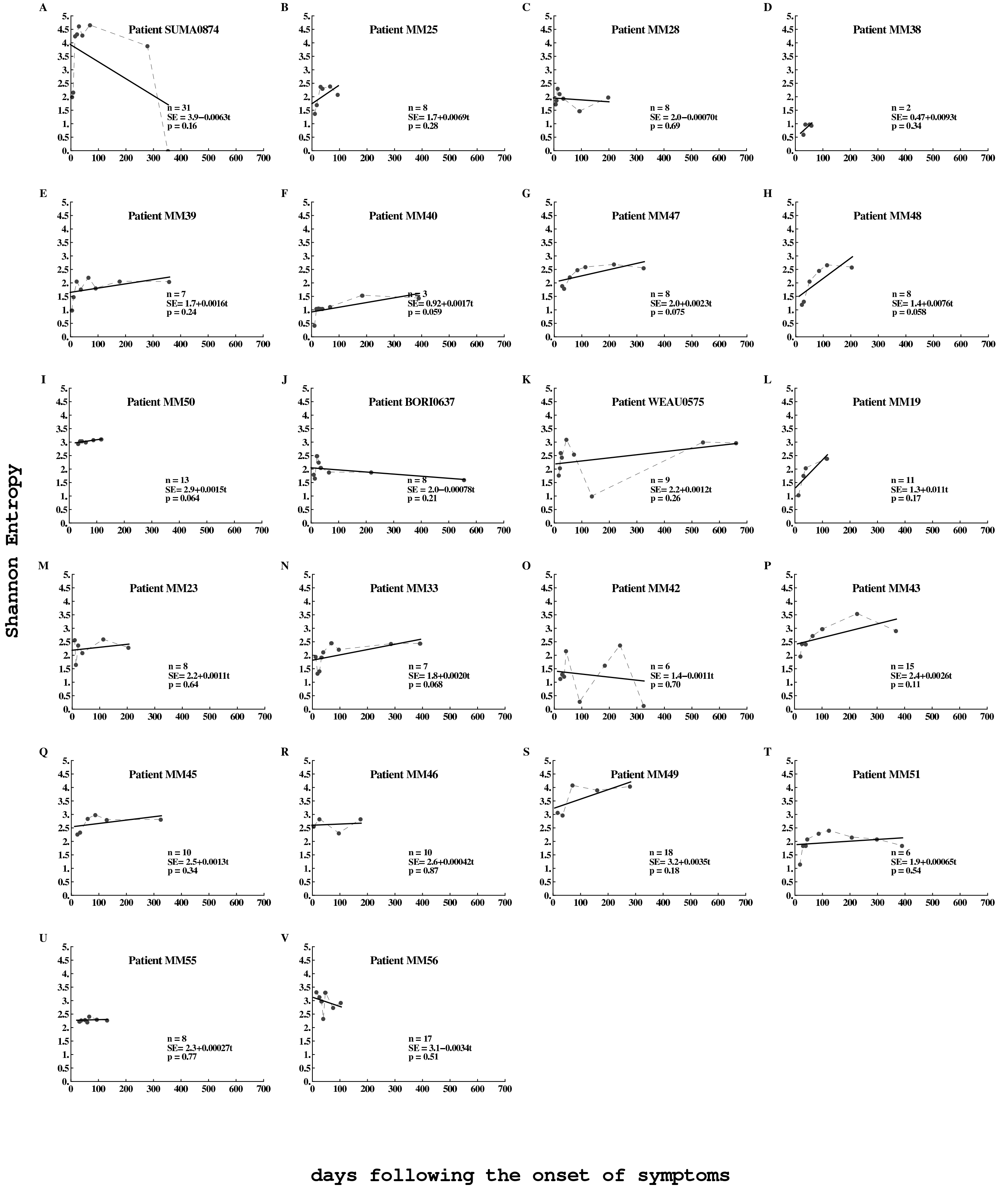

Figure S9: Kinetics of SI (dashed line) and corresponding linear fitted curve (solid line) for all patients. No trends were statistically significant (p values from linear regressions are indicated on panels).

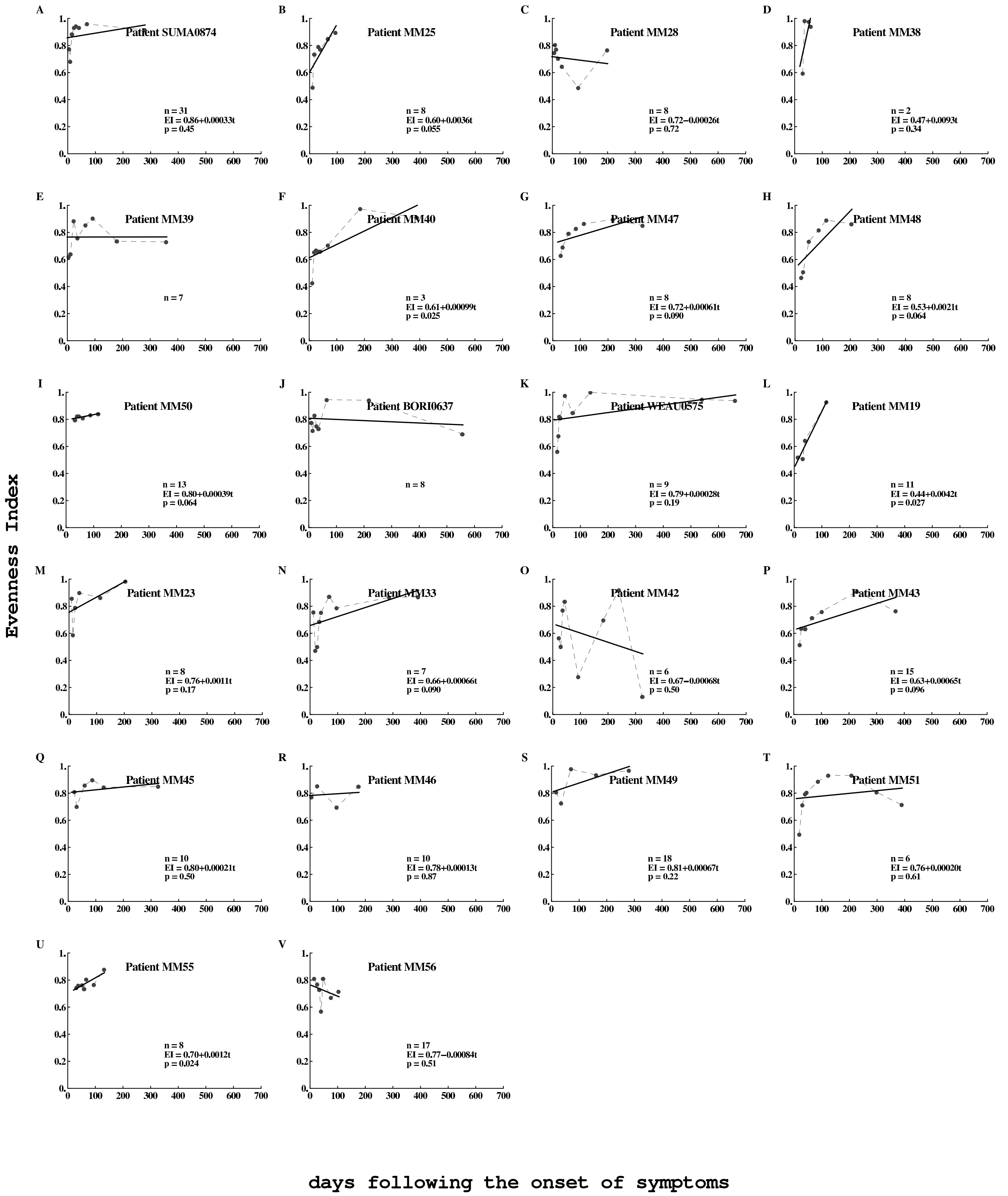

Figure S10: Kinetics of EI (dashed line) and corresponding linear fitted curve (solid line) for all patients. Two out of 24 patients showed significant increase in EI over time while other trends were not significant (p values for linear regressions are indicated on the panels).

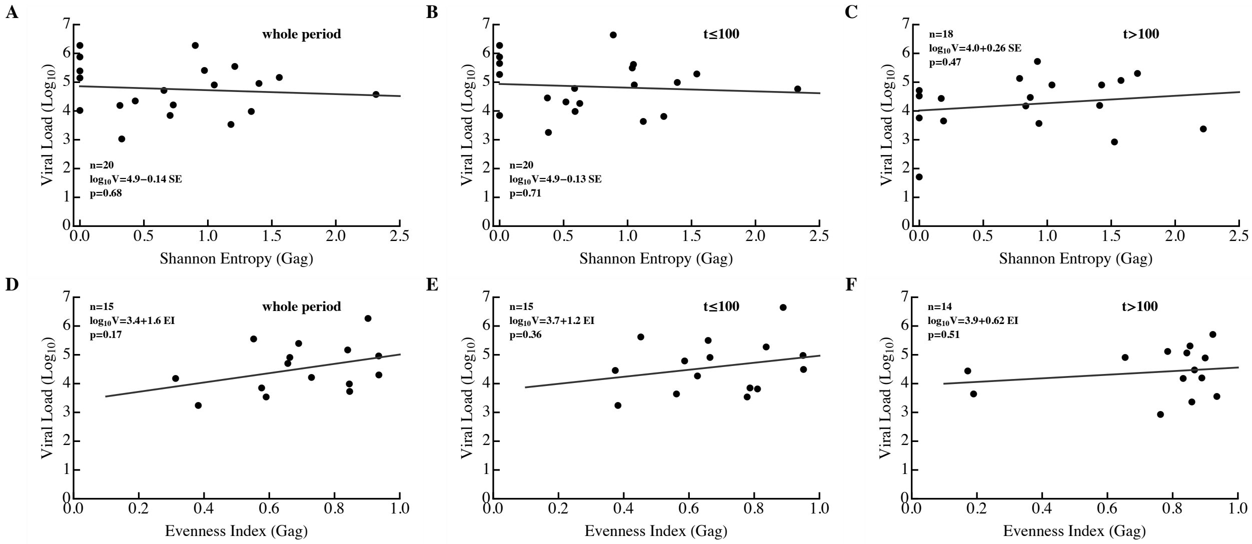

Figure S11: Variable correlations between viral load (V) and SE (A–C) or EI (B–F) of Gag-specific CD8$^+$ T-cell responses. Note a positive (but nonsignificant) correlation between viral load and breadth measured by EI, and positive correlation between breadth measured as SE and V for chronic infection ($t > 100$ days after symptom onset).

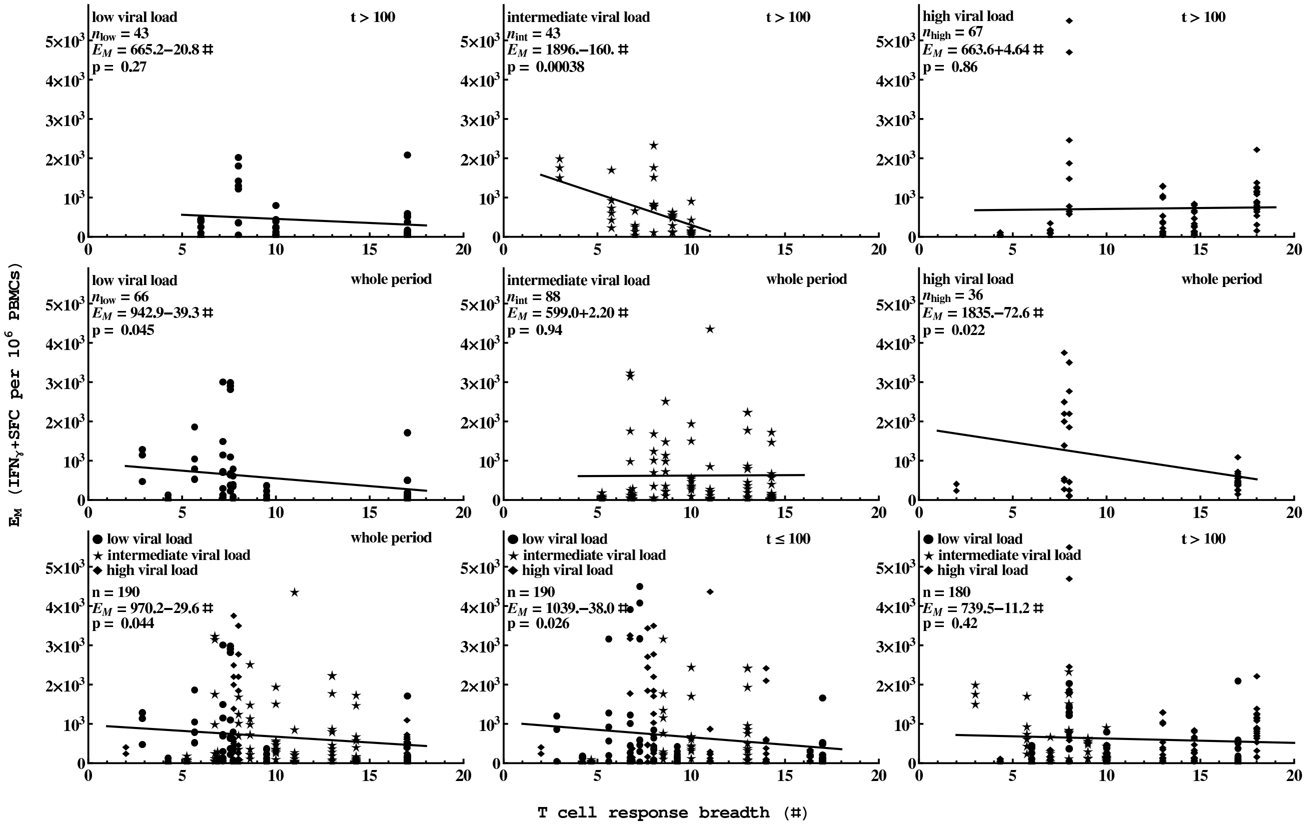

Figure S12: Correlation between number of T-cell responses and average size of T-cell response depends on viral load and time period since infection. Correlation between number of immune responses and average size of T-cell response is shown for chronic infection (top row) or for the whole time period (middle row) for different average viral loads. Bottom row shows correlation for all data at different time periods since infection. p values are from linear regressions; best fit equations are shown on individual panels. Some correlations are negative, indicating the presence of interclonal competition. Low, intermediate, and high viral loads were defined as described in Fig. 9.

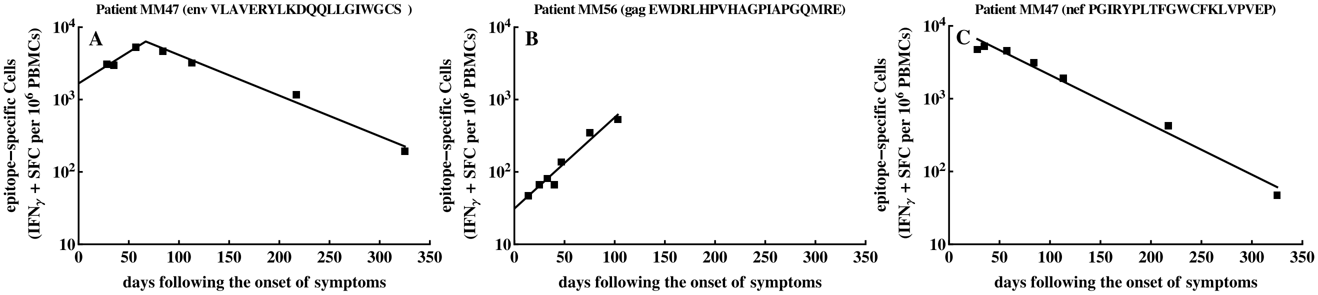

Figure S13: Examples of data on the kinetics of eptiope-specific CD8$^+$ T-cell responses and the predicted fits of the basic $T_{\rm on}$-$T_{\rm off}$ model eqn. (1) to these data. In all three examples there were no initial zeroes recorded so we set $T_{\rm on} = 0$ for simplicity.

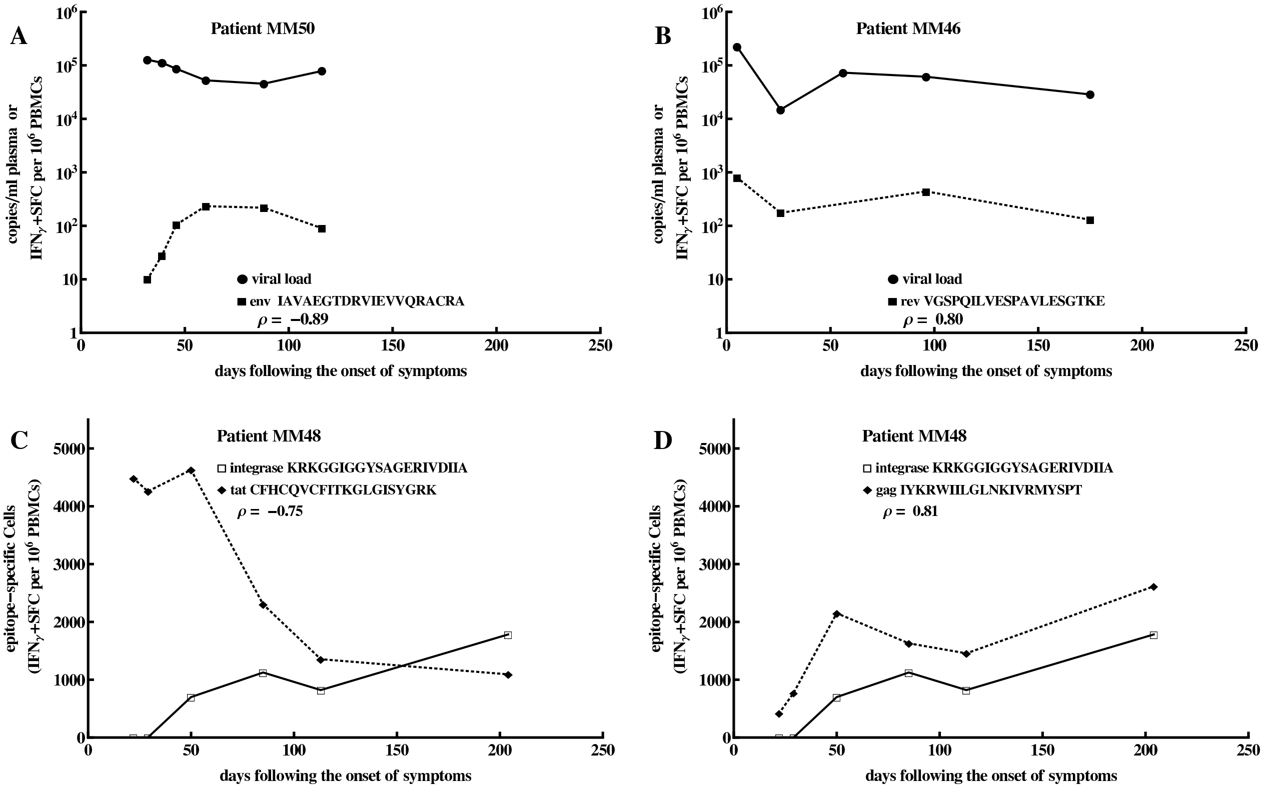

Figure S14: Examples of strongly negatively (A&C) and strongly positively(B&D) correlated viral load and epitope-specific $CD8^+$ T-cell responses or different epitope-specific $CD8^+$ T-cell responses. The correlation coefficients (ρ) were used to generate the histogram in Figures 5 or 8.

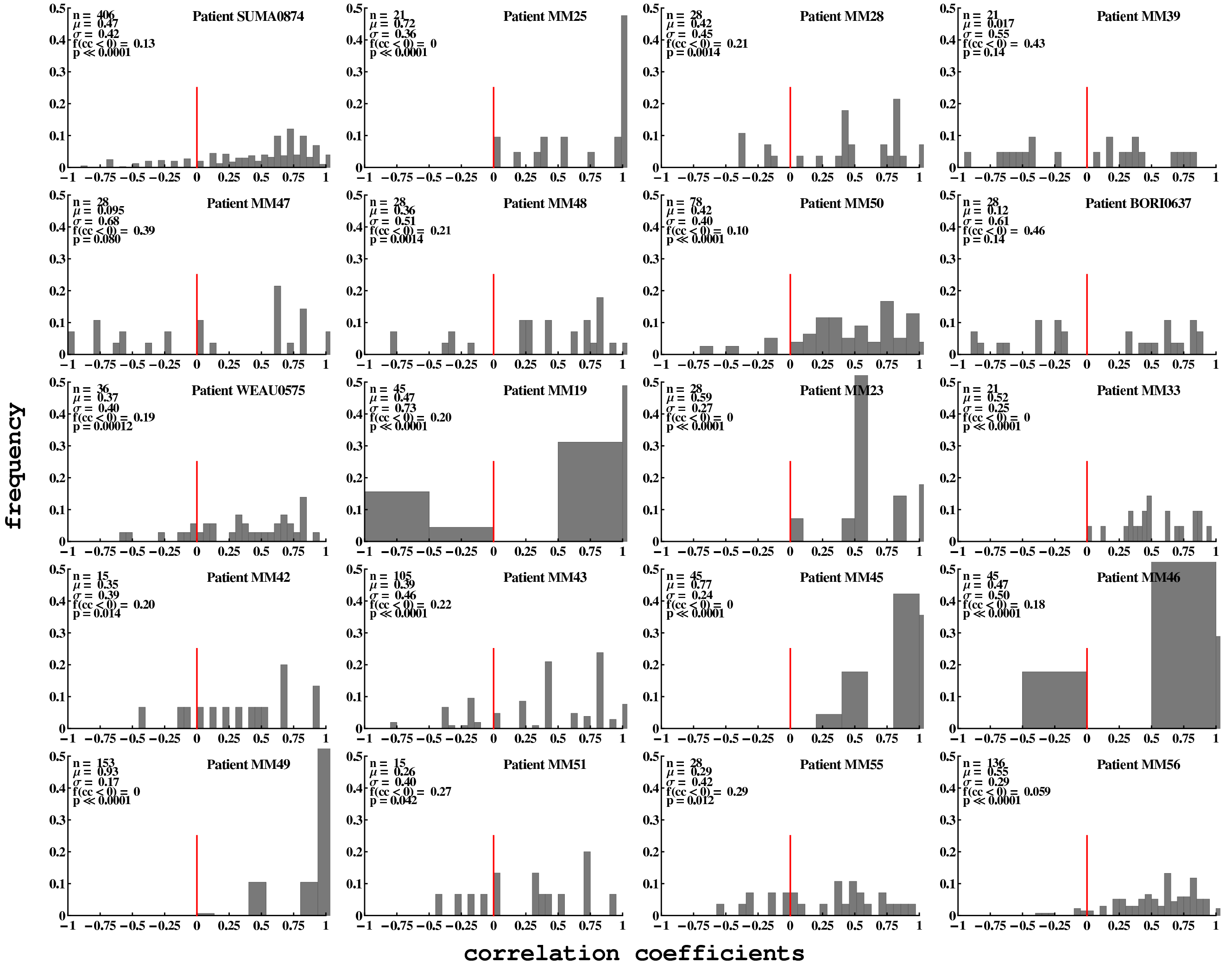

Figure S15: Detailed distributions of correlation coefficient (cc) between different epitope-specific CD8$^+$ T-cell responses (IRs) in different patients for $t \leq 100$ days after symptom onset (acute infection). Negatively correlated epitope-specific CD8$^+$ T cell responses were observed for nearly all patients, suggesting that interclonal competition between T cell responses specific to different HIV epitopes may occur in all HIV-infected patients.
